# SARS-CoV-2 infection: Understanding the immune system abnormalities to get an adequate diagnosis

**DOI:** 10.17305/bjbms.2020.5400

**Published:** 2021-10

**Authors:** Karen Medina-Quero, Omar Barreto-Rodriguez, Voltaire Mendez-Rodriguez, Anahí Sanchez-Moncivais, Ivette Buendia-Roldan, Leslie Chavez-Galan

**Affiliations:** 1 Laboratory of Immunology, Escuela Militar de Graduados de Sanidad, Mexico City, Mexico; 2 Instituto Nacional de Enfermedades Respiratorias “Ismael Cosio Villegas”, Mexico City, Mexico

**Keywords:** SARS-CoV-2, diagnosis, macrophage, lymphocyte, inflammation

## Abstract

COVID-19 is a current pandemic that emerged from China at the end of December 2019. Its aetiological agent is the novel coronavirus SARS-CoV-2. To identify biomarkers, diagnostic tools, treatments, or vaccines to decrease COVID-19 incidence and mortality, the scientific community is making extraordinary efforts to understand all aspects of this pathogen, from viral structure to the pathophysiology and immunological processes activated in the host. Diverse abnormalities have been found during SARS-CoV-2 infection both in lymphoid and myeloid cells. Such abnormalities can disturb the immune system function and cause a massive inflammatory response that impairs tissue function. This review discusses the close relationship between immune system abnormalities and the broad spectrum of clinical manifestations, including fibrosis, in the context of COVID-19 disease. Moreover, we have described the current strategies for COVID-19 diagnosis, and we provide a summary of the most useful clinical parameters to identify severe COVID-19 patients.

## INTRODUCTION

In late December 2019, an unexpected pandemic emerged from Wuhan, China; patients began to show pneumonia of unknown cause. On December 31^st^, 2019, the Chinese Centre for Disease Control and Prevention (China CDC) conducted an epidemiologic and aetiologic investigation, and described a novel coronavirus detected in these patients. The virus was designated as the severe acute respiratory syndrome coronavirus-2 (SARS-CoV-2), which is the etiologic agent of the coronavirus disease 2019, hereafter called COVID-19. It has been reported that SARS-CoV-2 is transmitted from person to person via droplet propagation [1].

COVID-19 has become a global health crisis. On January 31^st^, 2021, the World Health Organization (WHO) reported a worldwide total of 102,139,771 confirmed cases and 2,211,762 deaths attributed to COVID-19, with the Americas and Europe as the leading continents both in morbidity and mortality [2].

There is a growing body of evidence about the role of the immune response during SARS-CoV-2 infection. Reports suggest that a negative outcome of COVID-19 patients is strongly associated with an excessive production of pro-inflammatory cytokines, called “cytokine storm”. This effect is characterised by the uncontrolled regulation of the host immune response.

Only through the united efforts of the scientific community to create initiatives that seek development of vaccines, diagnosis tests and treatments accessible to everyone, we will be able to establish an adequate fight to COVID-19 and prevent SARS-CoV-2 transmission.

### What is SARS-CoV-2 and how it enters the cells?

SARS-CoV-2 genome sequence was obtained from bronchoalveolar lavage fluid samples and cultured isolates of COVID-19 patients. The genome is encoded in a single-stranded RNA fragment ~30 kb long and shares 79.6% and 96% sequence identity with SARS-CoV-1 and bat coronavirus (CoVs), respectively, at a whole-genome level [3,4].

**FIGURE 1:**
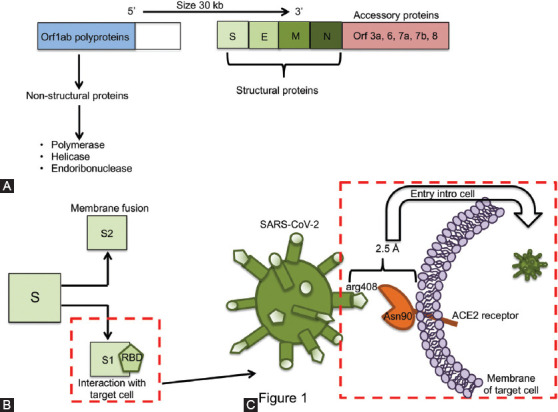
Genome structure and infection pathway of SARS-CoV-2. A) Near the 5’ end two thirds of the genome encode orf1ab polyproteins, which are non-structural proteins (polymerase, helicase, endoribonuclease); while the near 3’ end one third of the genome encodes structural proteins: spike (S), envelope (E), membrane (M), and nucleocapsid (N). Additionally, SARS-CoV-2 contains genes encoding accessory proteins (ORF3a, ORF6, ORF7a, ORF7b, and ORF8). B) During infection, the S protein is cleaved into S1 and S2 subunits; S1 subunit contains the receptor-binding domain (RBD), which is responsible of the interaction with the target cell, while S2 is responsible for membrane fusion. C) S1 (RBD region) from SARS-CoV-2 interacts with the ACE2 receptor expressed on a target cell. The interaction establishes a large binding interface between the amino acids Asn90 of ACE2 and Arg408 of the RBD core, forming a 2.5 Å crystal structure. Such structure favors the entry of SARS-CoV-2 into the cell.

SARS-CoV-2 genome structure is similar to other CoVs. Next to the 5’ end, more than two thirds of nucleotides encode ORF1ab polyproteins, which are non-structural proteins such as RNA-dependent RNA polymerase, helicase, and endoribonuclease. The remaining third, close to the 3’ end, encodes structural proteins including spike glycoprotein (S), envelope (E), membrane (M), and nucleocapsid (N) proteins. Additionally, SARS-CoV-2 genome contains accessory proteins, encoded by ORF3a, ORF6, ORF7a, ORF7b, and ORF8 genes (Fig. 1A) [3,5].

During SARS-CoV-2 infection, the trimeric S protein is cleaved into S1 and S2 subunits. The S1 subunit contains the receptor-binding domain (RBD), which interacts with the peptidase domain of angiotensin-converting enzyme 2 (ACE2) for entry into the target cell, whereas S2 is responsible for membrane fusion (Fig. 1B) [6,7]. ACE2 is expressed in the endothelium and can be secreted through proteolytic cleavage by the tumour necrosis factor-alpha convertase enzyme (TACE, also called ADAM17). The function of ACE2 is to catalyse the cleavage of Angiotensin (Ang) I to Ang1-9 by removing a single C-terminal leucine residue [8,9].

Reports indicate that the SARS-CoV-2 S protein interacts with ACE2 to mediate viral entry into the cell. S1 (RBD region) is essential for interaction with the ACE2 receptor, given that an extensive binding interface is established by a hydrogen bond between the Asn90 of ACE2 and Arg408 of the RBD core, forming a 2.5 Å crystal structure (Fig. 1C) [10,11].

SARS-CoV-2 and SARS-CoV display differences in the loop of the ACE2-binding ridge; SARS-CoV-2 has a higher affinity for receptor binding. SARS-CoV contains a three-residue motif proline-proline-alanine allowing a sharp turn, whereas SARS-CoV-2 contains a four-residue motif glycine-valine/glutamine-glutamate/threonine-glycine. The two relatively bulky residues and the two flexible glycine residues enable it to acquire a more compact conformation and get closer to ACE2 [10,11].

Previous reports have shown that ACE2 is abundantly expressed in lung tissue, and that it plays a beneficial role against lung disease [12]. However, it is essential to note that ACE2 expression in blood or airway tissue is elevated in cigarette smokers, patients with chronic obstructive pulmonary disease, hypertension, and asthma, when compared to healthy populations. Interestingly, children have significantly lower ACE2 expression than adults [13,14]. The information regarding SARS-CoV-2 structure or binding site in the host cell, may help decipher pathogenesis, design adequate therapeutic strategies or identify targets for vaccines. For instance, reports have shown that hospitalized COVID-19 patients develop antibodies that can recognize both S and N proteins, but 90% of the SARS-CoV-2 neutralizing humoral response specifically targets the RBD region [15].

### What is acute respiratory distress syndrome (ARDS)?

Clinical studies suggest that COVID-19 patients develop a robust inflammatory response characterised by an immune dysregulation which in turn, causes a cytokine storm [16]. Bronchoalveolar lavage (BAL) from COVID-19 patients show upregulated: a) chemokine genes such as CCL2 and CXCL8, which function as chemoattractants for inflammatory monocytes and neutrophils, respectively, and b) classical pro-inflammatory cytokine genes such as IL1RN and IL1B [17].

COVID-19 patients show a common complication of severe viral pneumonia called acute respiratory distress syndrome (ARDS). Reports suggest that 15.6% of COVID-19 patients with severe pneumonia develop ARDS, which is the most severe complication during SARS-CoV-2 infection [18]. ARDS is not a disease, it is a clinical condition defined as an acute respiratory failure that occurs in response to pulmonary insults and even though it was described for the first time in 1967, it remains as a major clinical problem worldwide [19]. Viral pneumonia is one of the most common clinical disorders associated with ARDS.

Huppert L.A *et al*. have carried out an extensive review of ARDS pathogenesis [20]. Microbial products bind to lung epithelium and alveolar macrophages wich induce an acute lung injury caused by dysregulated inflammation. Additionally, leucocyte signals destabilize vascular endothelial cadherin bonds, increasing endothelial permeability and accumulation of alveolar fluid; consequently, there is increased capillary permeability and pulmonary oedema formation. ARDS pulmonary oedema fluid contains high levels of pro-inflammatory cytokines including interleukin (IL)-1b, IL-6, IL-8, TNF, and transforming growth factor-b1 (TGFβ1) [20].

One of the main functions of the inflammatory response is to neutralize pathogens; nonetheless, excessive inflammation is detrimental to the host since it can result in a decline of lung tissue function. Regarding this, it is well known that the pro-inflammatory cytokine IL-6 plays an important role in the pathogenesis of the cytokine storm, and studies have revealed a correlation between high levels of IL-6 and severe status of COVID-19 [21].

At present, it is not completely clear how IL-6 favours the development of the cytokine storm. Recent evidence has suggested that N and S proteins of SARS-CoV-2 induce IL-6 secretion by human monocytes and macrophages, and that IL-6 inhibits Th1-dependent immunity and stimulates the presence of pro-inflammatory Th17 lymphocytes [22]. Other authors have suggested that IL-6 trans-signalling induces a robust inflammation circuit, which promotes the activation of the coagulation cascade and plasminogen activator inhibitor-1 (PAI-1). Moreover, plasma from severe COVID-19-ARDS patients exhibit high IL-6 and PAI-1 levels and the IL-6 trans-signalling/PAI-1 axis has been related to endothelial dysfunction [23,24].

### Phagocyte cells system: key players of the innate immune response

The phagocyte system (PS) includes dendritic cells, macrophages, and neutrophils, which may intake pathogens and particles in order to eliminate them [25]. Phagocytosis is a fundamental process that is necessary to activate the adaptive immune response through the presentation of peptide antigens, which are bound to specialized molecules called human leucocyte antigen (HLA).

Neutrophils are polymorphonuclear and highly motile cells and are the most abundant circulating leucocyte type. Neutrophils originate from hematopoietic stem cells (HSCs), which after several maturation steps, differentiate into fully mature segmented neutrophils. As recently discussed by De Filippo [26], a pool of mature neutrophils is retained in the pulmonary capillaries and studies suggest that they serve as a first line of defence in the lung. Apparently, this is beneficial since the presence of neutrophils that are already near, or inside the local environment, may increase the speed of the immune response, and help to maintain homeostasis; however, in the context of ARDS and some chronic lung diseases, excessive accumulation of neutrophils is associated with disease progression.

Lung macrophages are essential to maintain the homeostasis within the lungs. These macrophages are divided into a) alveolar macrophages: abundant in the alveolar space, originated from foetal monocytes and capable of self-renewal and, b) interstitial macrophages: originated from circulating monocytes and recruited into the lung after an insult; however, this is a transitional state because interstitial macrophages undergo a posterior differentiation process into monocyte-derived alveolar macrophages [27,28].

Although there are macrophages that are self-renewed, the major source of macrophages is circulating monocytes. Human circulating monocytes are categorized according to surface marker expression (CD14 and CD16), and their ability to deliver pro-inflammatory cytokines and migratory properties. Currently, these populations are divided into ‘classical’ (CD14^++^CD16^−^) and ‘non-classical’ (CD14^+^CD16^++^) monocytes. The second subset is related to perpetuation of the inflammatory environment, as they release abundant pro-inflammatory cytokines [29].

### What do we know about neutrophils during COVID-19?

Since the first reports, COVID-19 patients have shown a decreased frequency of leucocytes, but a higher neutrophil-lymphocyte ratio (NLR). Severe COVID-19 patients admitted to intensive care unit (ICU) show a higher frequency of neutrophils than severe patients not admitted to ICU [30]. Thus, the increased frequency of neutrophils is proposed as a prognostic factor for early identification of severe COVID-19 cases [31]. Autopsies have shown that there is an entrapment of high numbers of neutrophils within alveolar capillaries, and interestingly, these patients also showed an increased in neutrophil count in blood within 24 hours prior to the time of death [32].

Neutrophil extracellular traps (NETs) are structures of intracellular components released by activated neutrophils. The NET formation process is called NETosis, and although its function is to provide protection against infection, NETs have been implicated in pathological conditions as well [33]. Evidence suggests that in severe clinical forms of COVID-19, hyperactivity of the coagulation system engages in cross-talking with NETs, because SARS-CoV-2 is able to induce NETosis, increasing intracellular Reactive Oxygen Species (ROS). Although ROS promote pathogen-killing, these molecules also modulate the fibrinolysis system. Accordingly, reports have suggested that NETs play a major role in the pathogenesis of myocardial infarction in COVID-19 [34,35].

Neutrophils release neutrophil serine proteases (NSPs), which are related to ARDS development, however, COVID-19-ARDS patients do not show a different profile in NSP expression [36]. Nevertheless, moderate to severe clinical forms of COVID-19 show increased amounts of circulating extracellular DNA (ecDNA), which is considered a marker of inflammation and NET formation and interestingly, anti-SARS-CoV-2 IgA2 production correlates with ecDNA presence in severely ill patients. It is important to note that IgA2 stimulates pro-inflammatory effector functions in neutrophils [37].

Neutrophil activities are considered a cornerstone to favour the cytokine storm, thus neutrophils could be a major target for the development of therapies against SARS-CoV-2. In this regard, exogenous administration of recombinant DNase-1-coated nanoparticles or melanin-like nanospheres have been proposed to reduce ecDNA levels, neutrophil counts, and NETosis, ultimately modulating inflammation and lowering COVID-19 mortality [38,39].

### What do we know about monocytes during COVID-19?

A recent analysis has divided circulating monocytes from COVID-19 patients in 3 groups: classical, non-classical, and a third group of CD16^low^ monocytes, known as transitional monocytes. Authors suggest that all monocyte subsets are increased during COVID-19, and that a high frequency of monocytes correlates with higher levels of blood oxygenation. Interestingly, the reduction of non-classical and transitional monocyte frequency is associated with increased expression of IL-6. This reduction may be associated with the selective recruitment of these populations to the lung [40]. These results suggest that inflammation is transitional and non-classical monocytes preferentially migrate from blood to lungs in patients with severe COVID-19.

A report has indicated that critically ill COVID-19 patients have a reduced frequency of circulating monocytes that express HLA class II (HLA-DR), a molecule necessary for antigen presentation, compared to noncritically ill hospitalized COVID-19 patients [41].

In summary, BAL of severe COVID-19 patients are enriched with CCL2, the main chemoattractant of inflammatory monocytes, therefore migration of monocytes to the lungs increases, but these monocytes have decreased HLA-DR expression [16, 40, 41]. Altogether, this evidence suggests that SARS-CoV-2 induces monocyte dysfunction that consequently affects the function of lung macrophage subpopulations.

Evidence shows that SARS-CoV-2-infected monocytes under high-glucose conditions have an increased glycolytic capacity, which directly induces viral replication and pro-inflammatory cytokine expression [42].

Exploring the strategies to prevent monocyte over-activation or to use monocytes to monitor the critical status of patients could provide an alternative immunological intervention.

### What do we know about macrophages during COVID-19?

Post-mortem analysis of lung tissues has been relevant to establish important characteristics and to understand the role of macrophages in COVID-19, although several questions remain to be clarified.

At present, biopsy analysis has indicated that alveolar macrophages show high expression of Programmed Death-Ligand 1 (PD-L1) and ACE2 molecules. It has been established that the S protein of SARS-CoV-2 directly binds to macrophages by interaction with ACE2 [43]. Furthermore, it has been demonstrated that subcapsular lymph node and splenic marginal zone CD169+ macrophages express ACE2 and consequently, these macrophages are prone to SARS-CoV-2 infection [44].

Macrophages are one of the most important cell types that can induce the pro-inflammatory cytokine storm since it has been demonstrated that all COVID-19 patients with severe respiratory failure display ARDS or macrophage-activation syndrome (MAS). These complications induce an immune deregulation characterised by low HLA-DR expression on monocytes and macrophages, which is triggered by monocyte hyperactivation that promotes an excessive release of IL-6 [31,45].

Macrophages and monocytes are a source of several pro-inflammatory cytokines. For instance, a report has suggested that Interferon gamma-induced protein 10 (IP-10), monocyte-chemotactic protein 3 (MCP-3), and IL-1 receptor antagonist (IL-1RA), could be used as biomarkers associated with disease severity and fatal outcome [46]. High levels of IL-6, TNF and IL-8 are linked to macrophage activation and have also been correlated with the severity of the disease. Due to the current availability of monoclonal antibodies to block these molecules, they may be promising therapeutic targets for COVID-19 treatment [47].

### T cells: key players of the adaptive immune response

T cells are the leading players of the adaptive immune response and they are necessary to eliminate a broad spectrum of pathogens and malignant cells. In a simplified way, T cells can be divided into two groups: helper (CD4^+^) and cytotoxic (CD8^+^) T cells.

T cells mature in the thymus and posteriorly enter circulation into both in the vascular and lymphatic systems. T cells recognise HLA-bound antigens expressed on the surface of antigen presenting cells. Once the T cells are activated they carry out clonal expansion and perform different effector functions. CD4^+^ T cells are HLA class II restricted and differentiate into distinct subpopulations according to the cytokine profile delivered at the time of activation; whereas CD8^+^ T cells are HLA class I restricted and participate in the direct suppression of infected or malignant cells.

Within the COVID-19 context, Jarjou NN *et al*. [48] have done a thorough discussion on the regulation of the cellular and humoral immunity by the wide spectrum of T cell subpopulations. It focused mainly on the T cell maturation status to identify the optimal conditions to develop a rapid T cell expansion and differentiation into effector cells during the primary response, as well as the adequate generation of memory T cells, which is necessary to promote a more rapid and powerful response to reinfection.

To identify the differentiation status of T cells, it is relevant to know the optimal activation conditions of the immune response, which later would be fundamental for the development of vaccines or therapy schemes. For instance, in a viral respiratory infection it has been recently reported that naive CD4^+^ T cells (those that has not been exposed to an antigen) recognize a broad repertoire of antigens, from naturally processed to cryptic (structure hidden) peptides, whereas memory CD4^+^ T cells recognize just a few immunodominant antigens [49]. In this matter, Cooper RS *et al.*, [50] have recently reported that T cells isolated from individuals that have been infected with SARS-CoV-2 can be rapidly expanded *in vitro* and show a central memory phenotype. Authors have suggested that these T cells could also be used as therapeutic alternatives.

### What do we know about T cells during COVID-19?

Reports have demonstrated that the frequency of CD4+ and CD8+ T cells is reduced in COVID-19 patients. Interestingly, CD8^+^ T cells are dramatically reduced in severe cases compared to mild COVID-19 patients. This data is relevant because CD8^+^ T cells play a major role in suppression of virus-infected cells; thus, these cells are essential for controlling viral infections [31, 47]. Moreover, T cell function is necessary to establish a connection between cellular and humoral response. A report has suggested that the frequency of SARS-CoV-2-specific T cells is strongly correlated with the titres of neutralizing antibodies [51].

Although frequency of CD8^+^ T cells is decreased, the intensity of CD8 co-receptor expression, as well as granzyme B and perforin levels, is higher in COVID-19 patients than in healthy individuals and mild patients [52-54], suggesting that even though CD8^+^ T cell numbers are low in COVID-19 patients, these cells are highly cytotoxic. Recruitment of CD8^+^ T cells is not increased in the lungs, but there is an enrichment of activated CD8^+^ T cells and a depletion of dendritic cells [40]. This suggests that the antiviral function of these CD8^+^ T cells is impaired by a defect in the priming process. Another possibility is that virus-specific CD8^+^ T cells have an excessive activation, which can affect their adequate function. In this sense, CD8+ T cells from severe COVID-19 patients have been reported to display activation of IFN signalling that correlates with the disease severity, but in contrast, Yao C *et al*. identified that these CD8+ T cells (from severe COVID-19 patients) have a gene expression profile related to a deficiency in cytotoxic function [55].

In this regard, there is a growing body of evidence indicating that T cells display limited function as a result of exhaustion during SARS-CoV-2 infection. These T cells express immune-inhibitory molecules on their surface, such as programmed cell death protein 1 (PD-1), T-cell immunoglobulin and mucin-domain containing-3 (Tim-3), cytotoxic T-lymphocyte-associated protein 4 (CTLA-4) and the T cell immunoreceptor with Ig and ITIM domains (TIGIT).

Severe COVID-19 patients have a reduced frequency of multi-functional CD4^+^ T cells (producers of at least two types of cytokines) and apparently, this functional damage predisposes to severe disease [53]. Reports indicate that CD8^+^ T cells display excessive exhaustion, which is characterised by expression of PD-1, CTLA-4, Tim3 and TIGIT; but apparently the PD-1 and Tim-3 expression is increased mainly in patients that progress from prodromal to visibly symptomatic stages [53, 56]. Accordingly, lymphopenia, excessive T-cell activation and high expression of T-cell inhibitory molecules have been observed in lung tissue from COVID-19 severe cases [57].

The role of CD8^+^ T cells expressing PD-1 during SARS-CoV-2 infection is controversial. Recent reports have shown that IFN-γ-producing cells are lower among SARS-CoV-2-specific CD8^+^ T cells than those specific to influenza A virus; however, the proportion of IFN-γ-producing cells is mainly within the fraction of PD-1^+^ cells, suggesting that in the COVID-19 context, PD-1-expressing CD8^+^ T cells are not exhausted and these are functional cells [58]. The use of single-cell deep-immune profiling of BAL samples has allowed the evaluation of pseudotime trajectories of cellular lineages. Mild COVID-19 patients CD8^+^ T cells have shown expression of exhaustion markers, and that these are enriched halfway into their trajectories; however, these CD8^+^ T cells exhibit good effector functions. Nonetheless, in critical COVID-19 patients CD8^+^ T cells show evidence of inflammation-associated stress at the end of their trajectories [59].

In contrast, another report suggests that the frequency of exhausted SARS-CoV-2-reactive CD8^+^ T cells is increased and that these cells are less cytotoxic and inflammatory in mild COVID-19 patients when compared to severe COVID-19 patients. On the other hand, non-exhausted, SARS-CoV-2-reactive CD8^+^ T cell subsets (from severe patients) express transcripts related to co-stimulation and pro-survival mediated by NF-κB signalling [60].

Although the expression of exhaustion markers on T cells within the COVID-19 context is clear, the functional status of these T cells poses several questions regarding the diversity in the nature of CD8^+^ T cell response to SARS-CoV-2. More efforts are necessary to obtain a detailed understanding of the role of these exhausted CD8^+^ T cells during the anti-SARS-CoV-2 immune response.

The group of older COVID-19 patients is associated with a high mortality rate. The quantity of naïve T cells is typically compromised with increasing age. It has been speculated that the older COVID-19 patients have a low number of epitope-specific naïve T cells that could be primed. Accordingly, it has been proposed that the shortening of telomere length (the normal process during cellular senescence, which culminates in a cessation of replication) promotes the decline of T cell numbers and their exhaustion [61, 62]. Thus, if naïve T cells are the primary source to activate an adequate adaptive immune response, future research should include the development of strategies to enhance the naïve T cell pool in this group of patients and the role of telomerase could be helpful and should be studied deeply.

### Clinical characteristic and diagnosis of COVID-19

The status of immunological activation evaluated in laboratory studies, together with tomographic images, provide the necessary information to interpret the severity of tissue damage, pulmonary infiltrate, and level of pro-inflammatory cytokines. Altogether, these parameters are helpful to identify the status and evolution of COVID-19 patients. The knowledge of the basic immunological process that we have discussed above is fundamental to make a correct interpretation of clinical laboratory studies.

Some examples of how the basic information is related to the clinical parameters are: 1) the cytokine storm (for instance, high IL-6 levels in severe condition) is a result of an imbalance on T cells subsets, 2) the pro-inflammatory macrophage subpopulation is increased in severe but not in mild disease, 3) NETs generation promotes a hypercoagulation state and microvascular damage and, 4) ACE2 is expressed on the cell surface of several cells, including enterocytes which could explain clinical manifestations such as gastrointestinal complications.

In summary, understanding the implications of immune response for each of the symptoms of COVID-19 gives us a broader picture to design treatments and prevent cytokine storm and ARDS in critically ill patients. Below, we discuss the main clinical characteristics used for the diagnosis of COVID-19.

It is well established that SARS Cov-2 infection occurs from person to person through aerosols or droplets of fluids. The virus remains active up to 3 hours and on inert surfaces such as copper, cardboard, stainless steel, and plastic and it can remain viable up to 3 days, all of these facilitating the spread of the virus through contagion by contact with these surfaces [63].

The awareness of the incubation period length for SARS-CoV-2 has important public health implications. For example, this information is helpful to actively monitor, surveil, control, and model this disease. Reports indicate that the mean incubation period of SARS-CoV-2 is 5.1 days and infected persons may present symptoms within 12 days of contagion. Therefore, the current period of active monitoring recommended by the Centres for Disease Control and Prevention is 14 days following exposure [64].

The spectrum of clinical manifestations of COVID-19 is vast; however, main symptoms include dry cough, headache, fever, and fatigue, which can be accompanied by myalgias, arthralgias, loss of smell and sore throat, among others [65-68]. Although these are the most common symptoms associated with COVID-19, Table 1 shows the variations of symptoms and comorbidities in cohorts from different populations [17, 69-71].

**TABLE 1:**
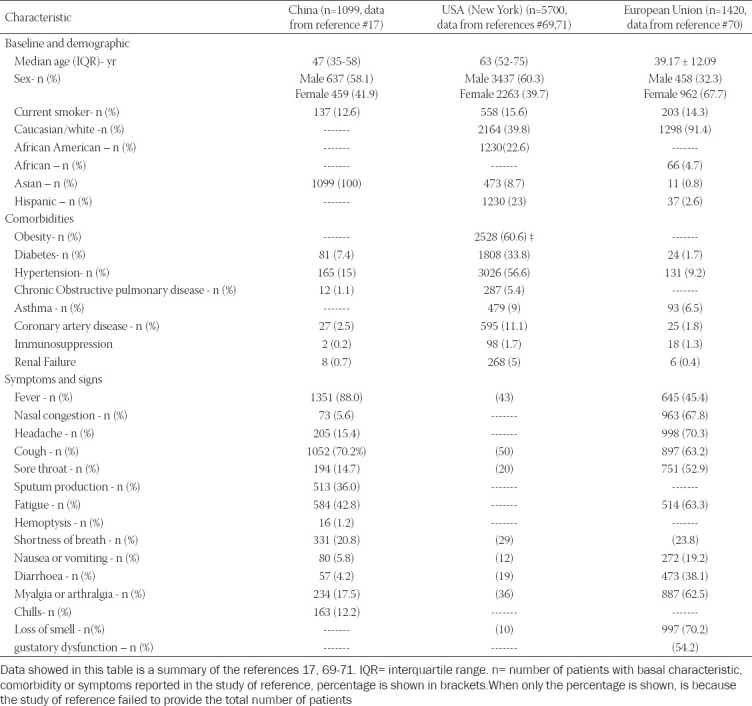
Clinical characteristics of patients diagnosed with COVID-19

The course of COVID-19 can be mild (usually no need for patient care), moderate (requires admission for care and typically presents pneumonia) or severe (requires intensive care and typically includes respiratory failure) [72]. Descriptive and retrospective studies have reported that severe COVID-19 status is related to different comorbidities such as diabetes, hypertension, obesity (body mass index > 30), chronic respiratory diseases, kidney disease, and immunosuppression status. Severe COVID-19 patients have a poor outcome, usually they require hospitalization and present persistent fever, dyspnoea, chest pain, signs of respiratory distress, and oxygen saturation of less than 92% [17, 69-71, 73].

The clinical follow-up of severe COVID-19 patients is summarized in Table 2; the recommendation of CDC about performing a chest CT-Scan, together with blood tests that include hematic biometry, blood chemistry, liver function tests, and analysis of ferritin, D-dimer, and troponin levels. These are useful as severity markers, as each one has a substantial impact on the outcome of COVID-19 patients [15, 17, 69, 73-76].

**TABLE 2:**
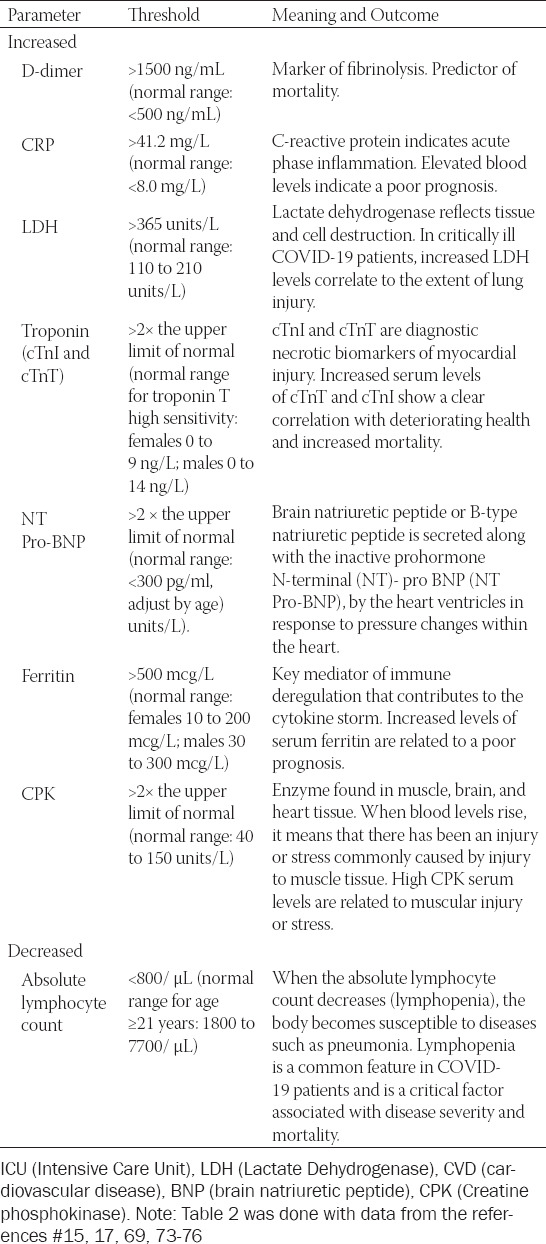
Clinical parameters associated with severe COVID-19

**FIGURE 2:**
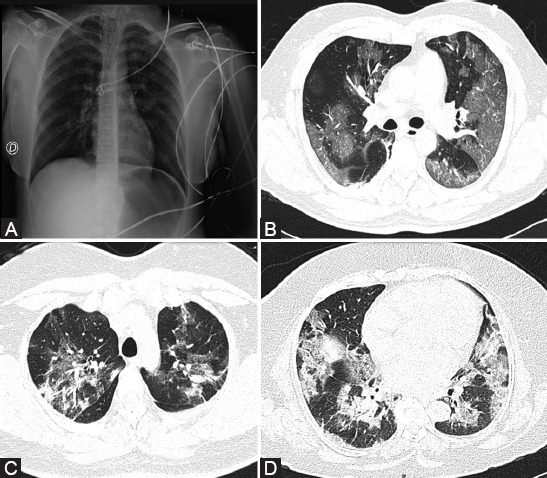
Classical images of X-Ray and CT-Scan from a female COVID-19 patient. A) Portable X-Ray with diffuse bilateral ground-glass patches. B) Chest CT-Scan with subpleural predominance ground glass sample (initial phase of the disease). C and D) Chest CT-Scan is showing a crazy paving and consolidation pattern (disease progression).

Chest CT-scanning has both diagnostic and prognostic value and shows an accuracy of 98% for the diagnosis of COVID-19 [77]. Classical images of a COVID-19 patient are shown in figure 2. Diffuse bilateral ground-glass patches are observed through X-ray imaging (Fig. 2A). In the early stages of the disease ground-glass patches of subpleural and basal are observed through a chest CT-Scan (Fig. 2B); whereas, during the progression of the disease a pattern of crazy paving or consolidation is observed (Fig. 2C and D). Furthermore, prognosis can be established according to the extent of lung damage, since patients that display more than 50% of lung damage must be hospitalized.

Gattinoni *et al*. [78] have proposed the existence of two identifiable COVID-19 phenotypes according to the pulmonary behaviour of the disease and have suggested the classification of patients with L (low) or H (high) phenotypes referring to the degree of elastance, ventilation to perfusion (VA/Q) ratio, lung weight, and lung cell recruitment.

Concerning the aetiological diagnosis, it is imperative to define the time of symptoms onset to determine which test is the best. Currently, the reverse transcription-polymerase chain reaction (RT-PCR) is considered the gold standard to identify SARS Cov-2 during the first 14 days of infection. Reports indicate that the RT-PCR test shows sensitivity variations and it has been suggested that the best time to perform this test is at the fourth day of symptoms onset, because of the viral load and the site of the sampling, among other factors. After 14 days from the onset of symptoms, the evaluation of IgG antibody levels by quantitative- (ELISA) or qualitative-methods (chemiluminescence) is useful [79-83]. Finally, a critical step in the clinical follow-up is the correct identification of the clinical phase of COVID-19 patients. Thus, both the period of viral replication and the inflammatory period should be considered, given that these would influence the therapeutic strategy [84].

Although the scientific community is continuously making efforts to understand the physiopathology of COVID-19, several questions are still unresolved, and their answers are necessary to control the spreading of SARS-CoV-2. Therefore, early recognition and appropriate treatment of the immunological complications is necessary to succeed in the fight against the virus.

### Fibrosis: another story to be included in the COVID-19 context

As previously discussed, COVID-19 severe illness may quickly progress to ARDS [85]. Recent reports have shown that the main risk factors for the development of ARDS are older age, neutrophilia and high levels of lactate dehydrogenase (LDH) and D-dimer, which simultaneously were associated with a fatal outcome. On the contrary, lymphocyte counts, CD8/CD4 T cell ratio, aspartate aminotransferase, prealbumin, creatinine, glucose, low-density lipoprotein, serum ferritin and prothrombin were not associated with mortality. Interestingly, the difference in mean levels of D-dimer between death and survival groups suggested that disseminated intravascular coagulation is the mayor cause of death and not ARDS [86].

In patients form the survival group that coursed ARDS, a major concern is the development of pulmonary fibrosis. In a cohort of 837 patients at four weeks post-discharge, 35 (4.8%) patients showed pneumonia with significant functional deficit. The treatment of these patients with corticosteroids was associated with a rapid and significant improvement [87]. However, in a different cohort of 48 mechanically ventilated survivors that were followed-up during 3 months after hospital discharge, ground glass opacities were reported in 41 (89%) cases and 31 (67%) cases showed signs of reticulation with fibrous bands either with or without obvious parenchymal distortion, bronchiectasis and bronchiolectasis [88].

There are different explanations for the development of fibrosis. For example, the ACE-2 receptor is considered to have a protective role in pulmonary fibrosis and this receptor also plays a critical role for the entry of the virus into cells. Other reports have suggested that due to SARS-CoV-2 binding to ACE2, the level of the receptor is decreased and consequently, pro-inflammatory and profibrotic effects are promoted [89]. Previous reports have shown that the N protein (of SARS-CoV) promotes expression of TGFβ in lung epithelial cells, causing fibroblasts to express PAI-1 and favouring the blocking of apoptosis to promote tissue fibrosis [90]. It is in this manner that reports propose that SARS-CoV-2 induces stress on the endoplasmic reticulum, and this could result in apoptosis and release of profibrotic molecules [91,92].

### Ethical statement

X-Ray and CT-Scan figures included in this review were obtained as part of a protocol authorized by the Ethics Committee (Protocol number C41-20) from the Instituto Nacional de Enfermedades Respiratorias Ismael Cosio Villegas. All procedures were performed in compliance with the 1964 Declaration of Helsinki.
